# Methodological Considerations and Evidence Interpretation in the Phase III Evaluation of the Absnow Biodegradable ASD Occluder

**DOI:** 10.1002/clc.70319

**Published:** 2026-05-11

**Authors:** Saad Arif, Hamna Bibi

**Affiliations:** ^1^ Ayub Medical College Abbottabad Pakistan

Dear Editor,

We read with interest the study by Fan et al. reporting 4‐year outcomes of the Absnow fully biodegradable atrial septal defect (ASD) Occluder in children [1]. The development of resorbable ASD closure devices represents a significant step forward, and the author should be commended for providing midterm clinical data in this emerging area. However, we believe several aspects of the study's framing may unintentionally overstate the strength and generalizability of the evidence.

First, the study is described as a “phase III clinical trial” that “confirmed” safety and effectiveness. However, the data come from a single‐center, single‐arm cohort of 10 carefully selected pediatric patients without a comparator device. In current device development, phase 3 studies are typically large, multicenter, and designed to provide confirmatory evidence for regulatory and clinical decision‐making [2]. Describing this small, uncontrolled series as a phase III may suggest a level of evidentiary strength that the study design cannot fully support. The findings may be more appropriately viewed as early clinical experience within an ongoing development pathway.

Second, the use of the term “acceptable” safety and effectiveness lacks a clear clinical context. Although early closure rates were favorable. The effective closure rate decreased to 70% at 4 years, with three patients developing significant residual shunts (≥ 4 mm) and two requiring re‐intervention with metallic devices. Without a comparator group or predefined performance benchmarks, it is challenging to determine whether these outcomes would be considered acceptable for routine clinical practice, especially given higher long‐term closure rates reported with the conventional Occluder in larger studies [3]. Providing a clearer performance context would help readers interpret these findings more accurately.

Third, generalizability appears limited to a highly selected patient population. The study primarily included children with anatomically favorable secundum ASDs (age ≥ 3 years, weight ≥ 10 kg, and adequate rims) treated at a specialized center with advanced imaging and procedural expertise. While the results support feasibility and reasonable short‐to mid‐term outcomes in this setting, extrapolation to more complex anatomies, borderline rims, older patients, or lower‐volume centers remains uncertain [4]. Clear emphasis on these boundaries would help avoid unintended overgeneralization.

Finally, post‐procedural elevations in lactate dehydrogenase (LDH), peaking at 3 months (mean 320 U/L) before a gradual decline, suggest potential device‐related myocardial stress or resorption‐mediated inflammation. However, the absence of long‐term clinical outcomes, image findings, or mechanistic discussion leaves the clinical significance undetermined [5].

In summary, addressing these limitations regarding trial designation, performance benchmarks, applicability constraints, and biomarker interpretations would enhance the manuscript's validity and underscore the imperative for prospective, multicenter investigations.

## Author Contributions

All authors have read and approved the final manuscript.

## Funding

The authors have nothing to report.

## Ethics Statement

The authors have nothing to report.

## Conflicts of Interest

The authors declare no conflicts of interest.

## Data Availability

No new data were created or analyzed in this letter.
